# Prediction-based association control scheme in dense femtocell networks

**DOI:** 10.1371/journal.pone.0174220

**Published:** 2017-03-22

**Authors:** Nak Woon Sung, Ngoc-Thai Pham, Thong Huynh, Won-Joo Hwang, Ilsun You, Kim-Kwang Raymond Choo

**Affiliations:** 1 Electronics and Telecommunications Research Institute (ETRI), Daejeon 34129, Republic of Korea; 2 Department of Information and Communications Engineering, Inje University, Gimhae 50834, Republic of Korea; 3 Department of Electrical Engineering, Tokyo University of Science, Tokyo 125-8585, Japan; 4 Department of Information Security Engineering, Soonchunhyang University, Asan-si 31538, Republic of Korea; 5 Department of Information Systems and Cyber Security, The University of Texas at San Antonio, San Antonio, TX 78249 United States of America; 6 School of Information Technology & Mathematical Sciences, University of South Australia, Adelaide, SA 5001 Australia; Beihang University, CHINA

## Abstract

The deployment of large number of femtocell base stations allows us to extend the coverage and efficiently utilize resources in a low cost manner. However, the small cell size of femtocell networks can result in frequent handovers to the mobile user, and consequently throughput degradation. Thus, in this paper, we propose predictive association control schemes to improve the system’s effective throughput. Our design focuses on reducing handover frequency without impacting on throughput. The proposed schemes determine handover decisions that contribute most to the network throughput and are proper for distributed implementations. The simulation results show significant gains compared with existing methods in terms of handover frequency and network throughput perspective.

## 1 Introduction

The small femtocell can be inexpensively deployed to improve the quality of service (QoS) in wireless networks [1]. Installation of low-cost femtocells is generally indoors or in hotspots (to facilitate communications with cellular networks via a wired backhaul or separate radio frequency backhaul channels) as these femtocells can significantly improve indoor coverage and capacity. This is due to their capability to cover corners where radio signal from macro base station (MBS) may be weak, and short-distance communications enable a higher signal-to-noise ratio (SINR). Massive femtocell base stations (FBSs) within a range of a MBS may extend the coverage to wider areas such as large office buildings. In such a scenario, a mobile station (MS) may move to the new position, then associating with a new FBS to maintain connectivity. In another scenario, an active MS associates with a FBS to start a data session, which will be terminated when it is finished. In both scenarios, frequent associations or re-associations, or handovers are necessary to provide seamless service to the MS [2, 3]. Thus, the MSs need to make appropriate and informed association decisions.

Suffice to note that the ability to make an informed association decision is critical in the reliability of mobile femtocell networks. For example, a handover procedure introduces short-term service disruptions of MSs [4] (e.g., 50 milliseconds in WiMAX—see [2]). Therefore, one would prefer to have an association decision involving minimal handover decisions (i.e., a minimum handover frequency is preferred to guarantee service continuity). For example, by associating to the FBS which provide longer availability to an MS, the MS may reduce the handover frequency in a time period. In addition, the association decision significantly affects the instant throughput achieved by the MS. FBSs close to the MS or FBSs having association with a small number of MSs may provide better link quality than the others [3]. Unsurprisingly, association control has been investigated to address handover latency [4], handover frequency [5] and achievable throughput [6].

In particular, in the design of densely deployed femtocell networks, we need to address both handover frequency and service disruption as the small cell size of the FBS results in more frequent handover to moving MSs [7]. We remark that while handover latency caused by the scanning time and association periods locally affects each MS [4], co-related handover frequency and achievable throughput have effects on the network-wide performance. Specifically, the association schemes to maximize only throughput may result in frequent handovers and consequently, frequent service disruptions. On the other hand, the association methods to minimize only handover frequency may consequently lead to low MS throughput since the MS may seek to remain connected to the current FBS regardless of achievable throughput.

In this work we quantify effective throughput as the maximum achievable throughput excluding the service disruption periods caused by handover. Therefore this effective throughput can be regarded as a universal objective in MSs’ association control. Furthermore, we seek to determine an optimal association control policy from both handover frequency and achievable throughput perspective. This would allow us to improve the universal effective network throughput. In our approach, we assume that mobility information (e.g., mobility patterns or information of MS location prediction) is available. The optimal handover decision is determined by the association control policy. We remark that the research described in this work extends our earlier published work [8]. For example, Section 5 presents the additional features in the proposed association control policy. We have also extended the evaluations of the extended system under different parameters.

Our contributions in this research are as follows:

1. We investigate association control policies to consider their issues in femtocell networks, namely: handover frequency and achievable throughput (Section 4). We introduce offline problems of optimal association control for network-wide throughput under availability of the predicted mobility of MSs. An approximate offline algorithm that considers the problems in shorter time scale may reduce computational complexity. From the approximate offline policy, we show how to derive the online policy incorporated with the mobility information. This online algorithm requires only a small amount of information from neighbor base stations (BSs) to make association decisions.
2. We discuss relevant features of the proposed association control policy and present the handover algorithm (Section 5). We demonstrate using simulations (Section 6) that the proposed algorithm presents a significant performance gain in comparison to traditional methods, when error margin in mobility prediction is up to 40% of coverage of an FBS.

## 2 Related work

Studies on association control for network-wide performance have been extensively investigated under different environments, e.g., wireless cellular network [9, 10], WiFi 802.11 [4, 11, 12], with different names such as association control [9, 13] or handoff decision [4, 14], or load balancing [10, 11]. In [9, 13, 14], the authors formulate the handover problem as the dynamic control problem and solve it by using Markov Decision Process framework, in order to obtain better network-wide utility performance. However, solving these algorithms requires centralized computation and is NP-hard.

Besides, most of the simple approaches for handover decision is usually based on current received signal strength indication (RSSI) level [4] or the predicted RSSI [15] regardless of the achievable throughput or potential handover frequency that MS may suffer from. Under densely deployed femtocell networks, this causes serious problem to MS in terms of both handover frequency and throughput performance.

Since instant throughput of MSs and the BSs available to the MSs largely depend on the position of MSs, acquisition of the position of the MSs in a longer time period helps resource allocation improve network performance [16]. Specifically, by predicting achievable throughput at each BS, MS may avoid connecting to the BS that provides low throughput [14]. In addition by anticipating the future position, trajectory and velocity of the MS, the MS can avoid connecting to the BS in which it is likely to stay for a short period [17]. However, effects of frequent handover are not considered in these approaches.

Mobility information of MS has also been used to reduce handover frequency in small cell networks by considering handover costs and interruption time [5], expected sojourn time of MS at the neighbor BS [13]. Especially in [5], the anchor-local BS is proposed to reduce the interruption time of the handover process. The anchor-local BS acts as the reply node to forward data between the serving and the target femtocell BSs. The authors also analyze signaling costs and disruption time of the suggested method. However, the handover decision is based on the heuristic method, which cannot provide the optimal performance. In [13], the authors present the handover decision mechanism formulated as the Markov decision process. The objective function includes the expected sojourn time and the blocking rate. In these works the mobility of the MSs has been modeled as Markov chain where each state corresponds to a femtocell. Motivated by these researches, we also model the user mobility using the Markov chain. However, our objective function includes both interruption time and throughput of the user.

Throughput and handover frequency are both considered as the objective function in [12, 18]. In [18], the authors propose to maximize throughput and minimize total frequent handover. The extension of this approach is proposed in [12], where the communication overhead between MS and BS is also taken into the objective function. However, these approaches do not capture the impact of the mobility of the user on the performance of the networks. In these schemes, although frequent update of user association is critical in obtaining an enhanced utility performance, the authors do not consider the cost of service disruption followed by user’s periodical re-association.

Our work differs from the approaches discussed in this section. While most conventional approaches are based on a single decision attribute for handover such as handover frequency or throughput, our proposed method bases on both criteria in order to facilitate a more rational association decision making.

## 3 System models

**Fig 1:**
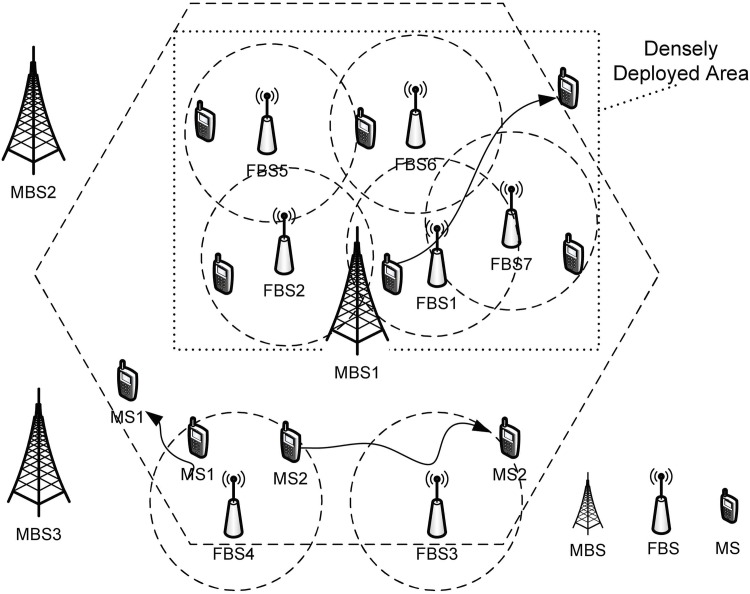
Femtocell networks.

In this research, we consider a downlink system of femtocell networks, comprising *M* BSs and *I* MSs (Fig 1). We denote by *m* ∈ *M* the index of BSs and *i* ∈ *I* that of MSs, respectively. Both FBSs and MBSs use the same technology such as 3GPP LTE or IEEE 802.16m/WiMAX and only differ from each other in signal strength and cell sizes. In our system model neighboring MBSs are assumed to be included in *M* BSs for simplicity. And we consider the downlink performance in our association control policy.

**Fig 2:**
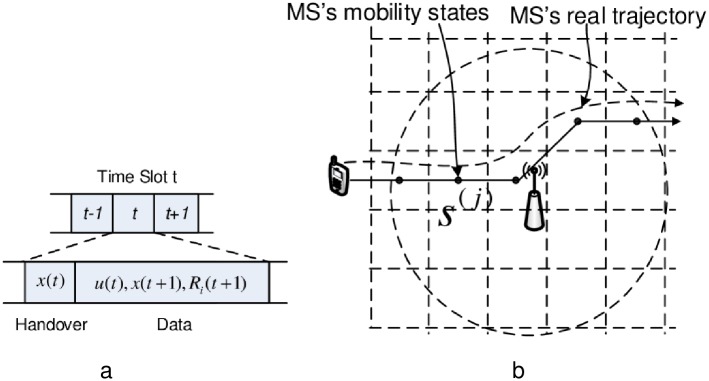
System model. a:Time slot structure. b: Example of mobility model.

Similar to [6, 13], we assume that time is slotted into equal time slots *t* = {0, 1, 2, …}. Each time slot comprises two sub-slots of handover and data (Fig 2(a)). At each sub-slot for handover, a set of available BSs *M*_*i*_(*t*) are acquired using the periodical scanning process. If handover is determined, MS *i* migrates to a new BS in *M*_*i*_(*t*). Now let *x*_*i*_(*t*) = {*x*_*i*, *m*_}_*i*∈*I*,*m*∈*M*_ denote a vector that describes MS *i*’s association state, where:

- *x*_*i*,*m*_ = 1 represents that MS *i* associates with BS *m*; and
- *x*_*i*,*m*_ = 0 represents that MS *i* does not associate with BS *m*.

We remark that MS *i* associates with only one BS *m* at time slot t; thus, this is described by $\sum_{m\in M}x_{i,m}=1$ which means that a hard handover is applied.

We denote by Ω_*i*_(*t*) a set of feasible association vectors of MS *i* for some available BSs *M*_*i*_(*t*) and Ω(*t*) = {Ω_*i*_(*t*)}_*i*∈*I*_, and we have *x*_*i*_(*t*) ∈ Ω_*i*_(*t*) and *x*(*t*) ∈ Ω(*t*).

*User Mobility*: The mobility of the user is modeled by the Markov process. As shown in Fig 2(b), we divide the geographical space as a set of zones, namely: *s*^(*j*)^ ∈ *S* = {*s*^(0)^, *s*^(1)^, …} and the location of MSs is specified by *s*^(*j*)^. Let *s*_*i*_(*t*) ∈ *S* denote the location of MS *i* at time slot *t*. The MSs moves around among these states *s*_*i*_(*t*) = *s*^(*j*)^ ∈ *S* and their mobility are determined by the Markov processes. Specifically, the successive state *s*_*i*_(*t* + 1) of MS *i* at *t* + 1 are governed by the following transition probability

$$\begin{array}{c} p_{i}^{\left(j,k\right)}=Pr\left[s_{i}\left(t+1\right)=s^{\left(k\right)}|s_{i}\left(t\right)=s^{\left(j\right)}\right]. \end{array}$$

MSs determine their next moving trajectories using their next mobility states, as well as their speeds.

It is important to note that due to the moving speed of each MS, its mobility states may be sparse, or dense. When the speed is high, the trajectory of the MS is discretized in spatially sparse states while when speed is low, the consecutive states may be nearby. In addition, the probability of MSs at mobility states at any time *t* can be computed from above one-step transition probabilities [19].

*Illustrative Example of Mobility Models*: This Markov mobility model can be used to describe different mobility patterns, e.g., straight line movements, random walks, and level of knowledge of the mobility patterns, e.g., unknown or known mobility patterns. For example, when an MS moves along the straight line, it is obvious that a sequence of mobility states are generated along the trajectory and the transition probability $p_{i}^{\left(j,k\right)}=1$ for all *j*, *k* belonging to the trajectory dependent on the speeds of the MS. As depicted in Fig 2(b), the MS trajectory is discretized into a sequence of mobility states along trajectory. In another case, the mobility patterns of the MS can be collected and predicted in use of a certain external predictor. The predicted information can also be stored in this Markov process [13].

*Throughput Model*: Under fixed transmission power at each BS, the achievable data rates of MSs provided by the associated BS depend on the received SINR and the scheduling policy of the BS. To estimate expected achievable throughput given by corresponding BSs at each mobility state, we make following weak assumptions: (1) Transmission power from each BS to the MS is fixed. Assigning equal power is near optimal in high signal to noise environment [9]. (2) An inter-cell interference management scheme, e.g., partial frequency reuse, is applied in this femtocell network so as the interference between BSs can be neglected. (3) A proportional-fair scheduling policy, which allocates channel resources to maximize log-utility function of accumulated rate of each MS, is used at the BSs.

We calculate the achievable data rate *R*_*i*_(*t*) of the MS *i* associated with BS *m* at the mobility state *s*_*i*_(*t*) as follows [9]:

$$\begin{array}{c} R_{i}\left(t\right)=\frac{h(y_{m}\left(t\right))}{y_{m}\left(t\right)}r_{i}\left(t\right), \end{array}$$ (1)

where

- $h\left(y\right)=\sum_{z=1}^{y}\frac{1}{z}$ occurs due to the fading gain. This gain is a function of the number of users *y* and measured directly at the BS.
- $r_{i}\left(t\right)=W_{m}\log\left(1+\frac{g\left(m,s_{i}\left(t\right)\right)P_{0}}{\eta (m,s_{i}\left(t\right))}\right)$ is the instantaneous throughput of MS *i* at the mobility state *s*_*i*_(*t*) = *s*^(*j*)^ ∈ *S* where *W*_*m*_ denotes the bandwidth of BS *m* and *P*_0_ is transmission power from BS *m*. *g*(*m*, *s*_*i*_(*t*)) and *η*(*m*, *s*_*i*_(*t*)) denote channel gain from BS *m* and additive noise when *s*_*i*_(*t*) = *s*^(*j*)^ ∈ *S*, respectively. The channel gain is dependent on the path-loss model, indoors or outdoors, and distances between the MS *i* and BS *m*.
- $y_{m}\left(t\right)=\sum_{i\in I}x_{i,m}\left(t\right)$ is the number of MSs associated with the BS *m*.

*Problem Statement*: Our objective here is to maximize the long-term effective throughput of the networks. The effective throughput is acquired by the data rates achieved by the MS as in Eq (1), but any service disruption period due to handover has to be excluded from this throughput. If MS *i* performs handover at the time slot *t*, its association state is changed to *x*_*i*_(*t* + 1), resulting in data rate *R*_*i*_(*t*) at data sub-slot *t* (i.e., (*x*_*i*_(*t* + 1) − *x*_*i*_(*t*))^2^ > 0). Let *ϵ* denote the disruption period due to handover between two BSs, which is a function of the number of time slots. In our system model, since the assessment for handover is periodically executed per time slot and its period is longer than the handover delay, we have *ϵ* < 1. From this, the effective throughput of MS *i* at time slot *t* + 1 is given as $\left(1-\frac{\epsilon }{2}{\left(x_{i}\left(t+1\right)-x_{i}\left(t\right)\right)}^{2}\right)R_{i}\left(t+1\right)$. Therefore, the association control problem is to seek an optimal policy which maximizes the total effective throughput of all MSs over *T* time slots:

$$\begin{array}{c} \max\;\;\;\;\;\sum_{t=0}^{T}\sum_{i\in I}\left(1-\frac{\epsilon }{2}{\left(x_{i}\left(t+1\right)-x_{i}\left(t\right)\right)}^{2}\right)R_{i}\left(t+1\right). \end{array}$$ (2)

## 4 Optimal association control

In this section, we construct association decision as an optimal control problem in use of the dynamic programming formulation. This formulation provides an insight into association decision and formal derivation of online policy for some specific and practical cases.

### 4.1 Dynamic programming for association control

We can regard the association control problem as a sequential decision problem with *T* stages, where at each stage the association vector *x*(*t*) is determined by the controller. This decision is bound with a reward defined by the effective throughput of MSs at that stage. Moreover, the reward of the successive stages also depends on the previous stage. The association control is formally defined as a dynamic programming problem, which consists of states and reward at each stage.

In our approach, a dynamic system is one whose each state is modeled as association state *x*(*t*). For each time slot, the controller makes association decision to generate the control action *u*(*t*) ∈ Ω(*t*) and forwards the system to the next association state. The next state *x*(*t* + 1) can be described as a function of *u*(*t*):

$$\begin{array}{c} x(t+1)=u(t). \end{array}$$ (3)

Eq (2) implies that the reward at association state *x*(*t*) can be quantified by the achievable data rate of MS *i* at its current mobility state. The latter is a sequence of random variables evolved by the one-step transition probabilities, when assuming that *s*_*i*_(0) = *s*^(*j*)^ ∈ *S* at time *t* = 0 is given according to MS location prediction. If $p_{i}^{\left(j\right)}\left(t\right)$ is the probability which MS *i* is at mobility state *s*^(*j*)^ at time *t*, we have $\sum_{s^{\left(j\right)}\in S}p_{i}^{\left(j\right)}\left(t\right)=1$ and describe the total expected reward as

$$\begin{array}{c} c\left(x\left(t\right),u\left(t\right)\right)=\sum_{i\in I}\sum_{s^{\left(j\right)}\in S}p_{i}^{\left(j\right)}\left(t\right)c_{i}^{\left(j\right)}\left(x\left(t\right),u\left(t\right)\right), \end{array}$$

where $c_{i}^{\left(j\right)}\left(x\left(t\right),u\left(t\right)\right)$ is the reward which MS *i* is expected to have at its mobility state *s*^(*j*)^. We calculate this reward at the state *s*^(*j*)^ using data rate $R_{i}^{\left(j\right)}\left(x\left(t\right),u\left(t\right)\right)$ as follows:

$$\begin{array}{c} c_{i}^{\left(j\right)}\left(x\left(t\right),u\left(t\right)\right)=\left(1-\frac{\epsilon }{2}{\left(x_{i}\left(t\right)-u_{i}\left(t\right)\right)}^{2}\right)R_{i}^{\left(j\right)}\left(x\left(t\right),u\left(t\right)\right). \end{array}$$

Throughput $R_{i}^{\left(j\right)}\left(x\left(t\right),u\left(t\right)\right)$ is closely related with the association state *x*_*i*,*m*_(*t* + 1) of MS *i* and instantaneous achievable throughput $R_{i,m}^{\left(j\right)}\left(x\left(t\right),u\left(t\right)\right)$ at each BS *m*. In addition by using throughput model described in Section 3, the instantaneous rate $R_{i,m}^{\left(j\right)}\left(x\left(t\right),u\left(t\right)\right)$ is calculated as follows:

$$\begin{array}{ccc} R_{i}^{\left(j\right)}\left(x\left(t\right),u\left(t\right)\right) & = & \sum_{m\in N_{i}\left(t\right)}R_{i,m}^{\left(j\right)}\left(x\left(t\right),u\left(t\right)\right)x_{i,m}\left(t\right),\\ R_{i,m}^{\left(j\right)}\left(x\left(t\right),u\left(t\right)\right) & = & h\left(\sum_{i\in I}x_{i,m}\left(t+1\right)\right)W_{m}\log\left(1+\frac{g\left(m,s^{\left(j\right)}\right)P_{0}}{\eta (m,s^{\left(j\right)})}\right). \end{array}$$

Now our stochastic programming problem is converted to deterministic one where the expected reward at each stage is determined by only control decision *u*(*t*) and state *x*(*t*). Let the control policy *π* be a sequence of consecutive control actions. Therefore a policy *π* is defined as a set of {*u*(*t*)}_*t*=0,1,…,*T*−1_ generated from a sequence of association control *x*(0), *x*(1), …, *x*(*T*). Finally we formulate our dynamic programming problem as the dynamic evolution of states mentioned in Eq (2) as

$$\begin{array}{c} \max_{\pi }\;\;\;J^{\left(\pi \right)}\left(x\left(0\right)\right)=\left\{c\left(x\left(T\right),u\left(T\right)\right)+\sum_{t=0}^{T-1}c(x(t),u(t))\right\}, \end{array}$$ (4)

where *x*(0) and *x*(*T*) are the initial and terminal states, and value function *J*^(*π*)^(*x*(*t*)) represents the total expected reward starting from *t* to time *T* under policy *π*.

Now, we focus on finding the optimal policy *π* to maximize the value function *J*^(*π*)^(*x*(0)). Let “*” denote the optimal states and control actions. We can rewrite the Problem (4) by using the Bellman equation as follows [20]:

$$\begin{array}{c} J_{t}\left(x^{*}\left(t\right)\right)=\max_{u(t)\in \Omega (t)}\left\{c\left(x\left(t\right),u\left(t\right)\right)+J_{t+1}\left(x^{*}\left(t+1\right)\right)\right\}. \end{array}$$ (5)

We can apply the backward induction to find the optimal control action of Problem (5). Starting from the terminal state x*(*T*), the optimal control action u*(*t*), and state x*(*t*) are computed recursively backward in time starting from *t* = *T* − 1 and stopping at *t* = 0. An optimal control policy π* is found out at the initial stage of *t* = 0 and consists of a sequence of control actions u*(*t*) (i.e. optimal association control actions at each time *t*).

### 4.2 Lookahead policy (approximate association control)

Apparently if *T* is large enough, the solution to Problem (4) becomes close to the optimal policy. However, a large *T* may make the size of parameters and system complexity very expensive. The *lookahead policy* [20] is an alternative approach to overcome these issues. This approach to problem solving utilizes a shorter time scale *T*_0_ < *T* ranging from *t* to *t* + *T*_0_, at every stage to decide a control decision *u*(*t*). Then this process is repeatedly executed before termination at *t* + 1. When the control policy follows the lookahead policy from *t* to *t* + *T*_0_, we denote by $J_{t,t+T_{0}}^{\left(L\right)}\left(x\left(t\right)\right)$ the sum of reward at *t* which is acquired by the *T*_0_-slot lookahead policy. The reward function $J_{t,t+T_{0}}^{\left(L\right)}\left(x\left(t\right)\right)$ at *t* is described as follows:

$$\begin{array}{c} J_{t,t+T_{0}}^{\left(L\right)}\left(x\left(t\right)\right)=\max_{u\left(\tau \right),\forall \tau =\left[t,t+T_{0}\right]}\left\{\sum_{\tau =t}^{\tau =t+T_{0}}c(x(\tau ),u(\tau ))\right\}. \end{array}$$ (6)

It should be noted that in Eq (6) the terminal state is unknown. The reward function $J_{t,t+T_{0}}^{\left(L\right)}\left(x\left(t\right)\right)$ at *t* is thus maximizing over all the possible terminal states. A way to solve Eq (6) involves enumerating all possible sets of terminal states, solving the dynamic programming for each state, and selecting $J_{t,t+T_{0}}^{\left(L\right)}\left(x\left(t\right)\right)$ which has the largest value among the sets. An alternative is that we enumerate all possible actions and outcomes over *T*_0_ and apply a brute force search strategy over the created action spaces.

However, at a shorter horizon the solution is not optimal but suboptimal. In Theorem 1 we prove the optimal gap of the solution obtained by the *T*_0_ lookahead policy. The optimality gap is proportional to the length of the lookahead algorithm *T*_0_. However, the increment of *T*_0_ might lead to the exponential growth of the state space and required information for the algorithm.

**Theorem 1**: *Optimal gap by the* *T*_0_-*lookahead policy is* $\frac{\epsilon T}{2T_{0}}r_{max}$.

*Proof.* For any *t* and any policy *π* we have following inequality:

$$\begin{array}{ccc} J_{t}^{\left(\pi \right)}\left(x\left(t\right)\right) & = & J_{t}\left(x^{*}\left(t\right)\right)-\frac{\epsilon }{2}{\left(x^{*}\left(t\right)-x\left(t\right)\right)}^{2}r_{max}\\ & \geq & J_{t}\left(x^{*}\left(t\right)\right)-\frac{\epsilon }{2}Ir_{max} \end{array}$$ (7)

where *r*_*max*_ is the maximum achievable rate of an MS and *I* denotes the number of MSs. Inequality Eq (7) is obtained by firstly re-associating from *x*(0) to x*(*t*) with cost of $\frac{\epsilon }{2}{\left(x^{*}\left(t\right)-x\left(t\right)\right)}^{2}r_{max}$ and then following a sequence of optimal controls by *J*_*t*_(x*(*t*)). Therefore we are able to re-write Eq (5) as follows:

$$\begin{array}{cc} J_{t}\left(x^{*}\left(t\right)\right) & =\max_{u\left(\tau \right)\in \Omega \left(\tau \right),\forall \tau =\left[t,t+T_{0}\right]}\left\{\sum_{\tau =t}^{\tau =t+T_{0}}c(x(\tau ),u(\tau ))\right\}\\ & \;+\max_{u\left(\tau \right)\in \Omega \left(\tau \right),\forall \tau =\left[t,t+T_{0}+1\right]}\left\{J_{t+T_{0}+1}\left(x\left(\tau +T_{0}+1\right)\right)\right\}\\ & =\max_{u\left(\tau \right)\in \Omega \left(\tau \right),\forall \tau =\left[t,t+T_{0}\right]}\left\{\sum_{\tau =t}^{\tau =t+T_{0}}c(x(\tau ),u(\tau ))\right\}\\ & \;+J_{t+T_{0}+1}\left(x^{*}\left(t+T_{0}+1\right)\right)\\ & =J_{t,t+T_{0}}^{\left(L\right)}\left(x\left(t\right)\right)+J_{t+T_{0}+1}\left(x^{*}\left(t+T_{0}+1\right)\right). \end{array}$$ (8)

The optimal value function $J_{t}^{\left(L\right)}\left(x\left(t\right)\right)$ at time *t* acquired by the *T*_0_ lookahead policy consists of twofold. While the association control follows the lookahead policy during first period from *t* to *t* + *T*_0_, it follows the optimal policy during second period from *t* + *T*_0_ + 1 to *T*. The first period results in the first term in the right side of Eq (9) and the second is led to *J*_*t*+*T*_0_+1_(x*(*t* + *T*_0_ + 1)), respectively. Note that since using lookahead policy, the state at *t* + *T*_0_ + 1 differs from optimality, which is x*(*t* + *T*_0_ + 1) as the case of inequality Eq (7). Thus, Formulas (7) and (8) lead us to the following conclusions:

$$\begin{array}{ccc} J_{t}^{\left(L\right)}\left(x\left(t\right)\right) & = & J_{t,t+T_{0}}^{\left(L\right)}\left(x\left(t\right)\right)+J_{t+T_{0}+1}\left(x\left(t+T_{0}+1\right)\right)\\ & \geq & J_{t,t+T_{0}}^{\left(L\right)}\left(x\left(t\right)\right)+\\ & & J_{t+T_{0}+1}\left(x^{*}\left(t+T_{0}+1\right)\right)-\frac{\epsilon }{2}Ir_{max}\\ & = & J_{t}\left(x^{*}\left(t\right)\right)-\frac{\epsilon }{2}Ir_{max}. \end{array}$$ (9)

Finally, the optimal gap by *T*_0_-lookahead policy is $\frac{\epsilon }{2}Ir_{max}$ for all MSs. On average, *T*_0_-lookahead introduces $\frac{\epsilon T}{2T_{0}}r_{max}$ for each mobility state.

Meanwhile, the backward induction algorithms has a computational complexity of *O*(*T*|*S*|^2^|Ω|) to calculate *u*(*t*) over *T* time slots. The complexity of *T*_0_-slot lookahead policy is small compared to the optimal policy Eq (5) because *T*_0_ ≪ *T*. However, Problem (6) has to be solved in a centralized manner, for example by a centralized BS, to decide association decision *x*(*t*). But this is not practical in mobile femtocell networks. In next section, we suggest suboptimal solutions to Problem (6) which approximate the reward function with a separable function of association state *x*_*i*_(*t*).

### 4.3 Online association decision

In this work the fading gain of $h(\sum_{i\in I}x_{i,m}\left(t\right))$ at BS *m* is approximated by the moving average of value *h*_*m*_(*t*). Therefore BS *m* monitors the number of its own associated users, estimates the fading gain, and calculates the moving average along time *t*. If MS *i* is at mobility state *s*^(*j*)^ and has association with BS *m*, the achievable data rate at each stage is written as:

$$\begin{array}{c} R_{i,m}^{\left(j\right)}\left(x\left(t\right),u\left(t\right)\right)=h_{m}W_{m}\log\left(1+\frac{g\left(m,s^{\left(j\right)}\right)P_{0}}{\eta (m,s^{\left(j\right)})}\right). \end{array}$$

As shown in above equation, now this rate relies only on which association states MSs are in. In addition it is intuitive that when MS *i* performs a handover, the reward of MS *i* and the total rewards of the serving and target BS are affected. This is because MS *i* disconnects from the serving BS and connects to the new target BS. Then we decompose Problem (6) into *M* smaller sub-problems for each MS. Each MS decides its association to maximize its rewards and total rewards of the serving and target BSs:

$$\begin{array}{ccc} J_{t,t+T_{0}}^{\left(L,i\right)}\left(x_{i}\left(t\right)\right) & = & \max_{u_{i}\left(\tau \right),\forall \tau =\left[t,t+T_{0}\right]}\\ & & \sum_{\tau =t}^{\tau =t+T_{0}}\left\{c(x_{i}\left(\tau \right),u_{i}\left(\tau \right))+\sum_{m\in M_{i}}\frac{h_{m}\left(t\right)}{y_{m}\left(t\right)+u_{i}\left(\tau \right)}r_{m}\left(t\right)\right\}, \end{array}$$ (10)

where $J_{t,t+T_{0}}^{\left(L,i\right)}\left(x_{i}\left(t\right)\right)$ is the total rewards obtained by the decision of MS *i*, *u*_*i*_(*t*) is the control action of MS *i* at time slot *t* to forward to next state *x*_*i*_(*t* + 1), and *r*_*m*_(*t*) is the total data rate of associated MSs at BS *m*. It is noted that the serving BS of MS *i* is included in the neighbor list of BS *M*_*i*_ but MS *i* is not included in *y*_*m*_(*t*). In these decompositions the expected throughput of related MSs is approximated by their current data rates. Thus, the total data rate of neighbor BSs is the only one which BS *m* needs for the approximation.

Finally our solution approaches to Problem (10) count on only MS *i*. This means that at each time slot each MS solves its problem and takes its own action independently. Under a short horizon *T*_0_ and a small size of available BSs Ω_*i*_(*t*) at each slot, Problem (10) can be solved by enumerating all possible sequences of association {*x*(*τ*)}_*τ*=*t*,*t*+*T*_0__ and picking the one that generates the one that is the solution of Eq (10).

The association control problem is actually finding consecutive association actions of MS *i* so as it maximizes the reward function $J_{t,t+T_{0}}^{\left(L,i\right)}\left(x_{i}\left(t\right)\right)$ which relies on the mobility distribution of MS *i* over the mobility space. In particular, effect of the speed of fast moving MS *i* is also reflected in reward function $J_{t,t+T_{0}}^{\left(L,i\right)}\left(x_{i}\left(t\right)\right)$. When the speed is very high, the reward function of association with FBSs is smaller than ones of association with MBSs. This is because there are fewer time slots for which MSs stay connected to the FBS than those for which MSs stay connected to the MBS. Thus, MSs would choose to connect to MBSs to maximize their rewards.

## 5 Further discussion

### 5.1 Special cases of association control problem

We now consider our proposed algorithm for two special cases of association control problems: (1) Zero handover delay and (2) constant throughput. In the former case, handover delay is considered nearly zero. In the latter case, throughput is constant over BSs and time. This case corresponds to the problem of minimizing handover frequency. In case of non-stationary MSs, following corollaries are helpful since they limit required information to compute optimal association in those special cases.

**Corollary 1** (Zero handover delay): *The proposed online policy is optimal when handover delay is zero*.

When handover delay is assumed to be zero (*ϵ* = 0), the lookahead policy achieves the optimal gap of zero. Problem (4) becomes:

$$\begin{array}{c} \max_{x(t)}\;\;\;c\left(x\left(t\right),u\left(t\right)\right). \end{array}$$

This implies that under handover delay of zero or if handover delay has a small effect, e.g., a very fast handover mechanism is applied, myopic policy is optimal. In this case, our algorithm maximize the reward at each stage. This case has been considered in [9].

**Corollary 2** (Constant throughput): *Let* *T*_1_ *is the sum of the remaining availability time* (*sojourn time*) *of the serving BS and the maximum availability time of neighbor BSs. The proposed policy is optimal when* *T*_0_ ≥ T_1_.

**Proof:** We denote by BS *m* the associated BS of MS *i* and BS *n* the neighbor BS which has the maximum availability time. Thus, to reach state *x*_*i*_(*t* + *T*_1_ + 1), MS *i* has to do handover twice: one from BS *m* to BS *n* and one from BS *n* to the next BS. This is because the availability of BS *n* is ended at *t* + *T*_1_. It is clear that this is the minimum number of handovers, or maximum reward since if MS *i* handovers to any BSs other than BS *n* it may have to handover at least two to reach *x*(*t* + *T*_1_ + 1).

The *T*_1_ lookahead policy also chooses to handover to BS *n* since it generates a reward of two handovers. Moreover, for any policy including the optimal policy that is generated by full horizon *T*, it needs no less than two handover to reach *x*(*t* + *T*_1_ + 1) as shown above. Thus, *T*_1_ lookahead policy is optimal for any horizon *T* > *T*_1_.

Corollary 2 provides the necessary optimality condition for the association control when every MS has constant throughput. It is useful to determine the amount of predictive information, i.e., how many time slots ahead are necessary, for the optimal association.

**Fig 3:**
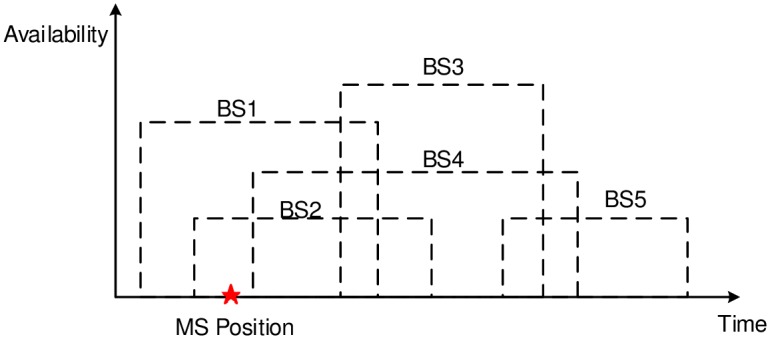
Example of availabilities of BSs.

Fig 3 provides an illustrative example of the Corollary 2. At MS Position, MS needs to know the availability time of BS4 which is the largest among the neighbors of the serving BS BS1. Thus, the decision of MS is optimal: it handovers to BS4 instead of BS2 to avoid an unnecessary handover, e.g., when doing handover to BS2, it has to handover again to BS3 or BS4. This is one unnecessary handover.

### 5.2 Complementary issues in femtocell handover

**Optimizing Neighbor BS List**: In dense femtocell networks, neighbor list Ω_*i*_(*t*) may be large. Consequently, periodical scanning and association period *ϵ* become large and the complexity of solving Eq (10) is high. We suggest a heuristic algorithm to reduce the size of this list. The algorithm removes the BSs that have insignificant expected rewards to the MS in a similar manner with the one in [21] for reduction of excessive scanning. We depict the importance of a BS by formulating its accumulated expected reward to a specific MS. For example, when an MS tends to move away from a BS, and it is at the edge of a BS, the accumulated expected reward of the BS would be small. The reward *C*_*i*,*m*_ during *T*_0_ slots is computed as follows:

$$\begin{array}{c} C_{i,m}=\sum_{\forall \tau =[t,t+T_{0}]}\sum_{s_{i}\left(t\right)=s^{\left(j\right)}\in S}p_{i}^{\left(j\right)}\left(t\right)R_{i,m}^{\left(j\right)}. \end{array}$$ (11)

Let *M*_0_ be the maximum number of neighbor BSs allowed in the shorten neighbor list. Only *M*_0_ BSs that have the largest expected reward *C*_*i*,*m*_ are selected to be scanned. Other BSs are omitted by the MS. Thus the shorten neighbor list size, i.e. *M*_0_, can be a kind of adjusting parameter for the association control of the MS. In general, a large *M*_0_ may provide better performance reward to the MSs but it requires more scanning time.

**QoS Guarantees**: QoS guarantees, e.g., guaranteeing the minimum achievable throughput of MSs, can be considered by incorporating extra constraints on selection of candidate BSs. When the throughput of the MS is guaranteed, that means it is greater than a threshold throughput parameter *R*_*th*_, it handovers to maximize the global rewards Eq (10). When the instant throughput of the MS is not satisfied, that means instant throughput is smaller than threshold parameter *R*_*th*_, it handovers to improve its throughput only.

Threshold parameter *R*_*th*_ is used to control the minimum achievable throughput of MSs. It is important to note that since this mechanism considers only instant throughput, it does not guarantee the throughput of every MS, but it helps each MS improve its throughput when the throughput falls low.

**Mobility Information**: Our model can be applied to both of two cases on the availability of the mobility information at MSs: (1) Mobility model and location prediction is available, (2) only location and trajectory are available.

- *Availability of mobility model and location prediction*: Under the availability of this information, MS *i* can update its current mobility state *s*_*i*_(*t*) and the probability of the future mobility states that it may visit. In this case, MS *i* performs the steps described above to calculate their optimal association.
- *Availability of location and trajectory prediction*: In this case, the probability of the future mobility state $p_{i}^{\left(j\right)}\left(\tau \right)$ is not provided. However, by using the information such as the current position, trajectory and speed of MS *i*, MS *i* can predict the mobility state *s*_*i*_(*t*) in the near future. We can assume that this information is valid for one time slot. Thus, the probability $p_{i}^{\left(j\right)}\left(\tau \right)$ at next time slot *t* + 1 can be generated roughly and performance reward with *T*_0_ = 1 can be calculated.

### 5.3 Handover algorithm

We now incorporate the proposed handover algorithm into the common handover procedures in femtocell networks [2, 7]. In a handover sub-slot, MSs and BSs process following steps:

- *Update mobility states*: MS *i* updates its current mobility state *s*_*i*_(*t*) based on position prediction and future mobility distribution $p_{i}^{\left(j\right)}\left(\tau \right)$ with *τ* = (*t*, *t* + *T*_0_)) based on one-step probability transition from mobility models. And MS *i* periodically reports its current mobility state and neighbor BSs’ RSSI measurements to the serving BS.
- *Obtain shortened neighbor list*: BS *n* broadcasts its diversity gain *h*_*m*_ and total data rate *r*_*m*_(*t*) to the neighbor BSs. BS *m* also generates the shorten neighbor list based on the current position and the expected reward of each MS by selecting *N*_0_ BSs that have the largest expected reward using Eq (11). Then BS *m* sends the shorten neighbor lists including the information on the diversity and total data rate to the associated MSs.
- *Decide Handover*: MS *i* calculates the optimal association decision for time slot *t* based on Eq (10). If handover is not necessary, it stays associated with the serving BS.
- *Scan the neighbor list and perform actual handover*: If handover is necessary, MS *i* scans for BSs on the shortened neighbor list and performs handover to the selected target BS.

Since an MS knows the diversity gains of only the neighbor BSs, the value of *T*_0_ should be considered to cover only the neighbor BSs.

## 6 Simulation

In this section the proposed online policy and the existing methods are evaluated and compared each other by varying performance criteria.

*Performance Metrics*: We evaluate association control methods over throughput, handover frequency and tradeoffs between two. For the proposed predictive association control in densely deployed femtocell networks, namely PADW, we also evaluate the ability to guarantee QoS, i.e. throughput, when QoS guarantees are applied following Remark 2.

*Comparative Methods*: PADW is evaluated in comparison with following existing association control methods:

- RSSI-based Handover Decision (RSSHD): This scheme is well-known and simple, and makes the handover decision based on the RSSI value [22]. Specifically, whenever the RSSI value of a new candidate BS is higher than that of the serving BS, an MS performs handover to the candidate BS. We evaluate this method using a hysteresis to decrease pingpong handovers. Hysteresis values ranging from 2 dB to 20 dB and default value of 4 dB are utilized since it is commonly adopted [23, 24].
- Sojourn Time-based Handover Decision (STHD): This location-based approach determines association control by estimating the expected sojourn time at the new candidate BS [25]. An MS has association with the candidate BS with the highest expected sojourn time, whenever it disconnects from the serving BS.
- Position-based Handover Decision (PHD): This method makes MSs associate with the closest BS [26]. We use a distance hysteresis to prevent pingpong effect. The hysteresis value ranging from 1 m to 30 m is utilized since the coverage of the femtocell is about 30 m.
- Throughput-based Handover Decision (TPHD): This scheme makes an MS handover to the BS with the highest expected throughput. We varied the population of the BSs and diversity gain function to estimate this throughput. Besides, a hysteresis is applied in order to avoid ping-pong effect [6, 9]. This hysteresis is a portion of the throughput and can be seen as a cost for each handover procedure. We evaluate this method with the hysteresis from 0.1 to 0.5 and a default value of 0.3 [6].

### 6.1 Simulation environment

**Fig 4:**
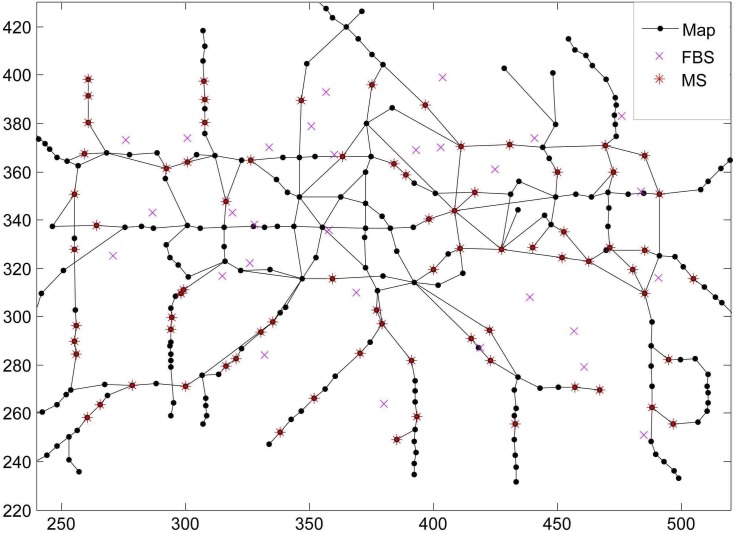
Simulation scenarios.

- *Network Scenarios*: For simulation WiMAX femtocell networks are configured to have 20 FBSs and 2 MBSs. We locate FBSs uniformly and the MSs randomly in a terrace (250, 500) x (250, 400). The coverage areas of BSs are circular with radius 280 m for the MBSs and 30 m for the FBSs. Since both of WiMAX MBS and FBS are a kind of OFDMA systems, MBSs and FBSs randomly assign the sub-channels to the MSs. Besides MBSs and FBSs are configured to transmit power up to 43 dBm and 13 dBm, respectively [27]. Note that all the BSs allocate equal power to each sub-channel. The radio propagation follows a popular pathloss model for WiMAX femtocell network: 34 + 40*log*(*d*) for the MBSs and 37 + 30*log*(*d*) for the FBSs. Furthermore the BSs apply the proportional fair scheduling to serve the MSs. And it is assumed that all MSs have infinity backlogged queue. The system bandwidth is 5 MHz and the time slot duration is 10 seconds. We run the simulation for 500 seconds, repeat simulations 50 times, and then take the averaged results.
- *Mobility Model*: Since we assume MSs to follow the pathway mobility model, they move along the connected path between two randomly selected points in a map [28]. Each point on the map is a mobility state. We use London metro map (Fig 4) where the MSs randomly choose their moving direction and speed on that path. We assume the MSs do not change this speed until the next point.
- *Location and Trajectory Prediction*: In our simulation we use the existing mobility method to predict the location and trajectory of MSs. Default error margins under 20% of range of a BS is assumed. Effects of this error rate on the performance of PADW are also evaluated in this section later.
- *Simulation Procedures*: We perform the simulations in a slotted time manner and at each time slot, the following steps:
  - Step 1: Update the MS location according to the mobility model
  - Step 2: Update the link quality and throughput for each MS
  - Step 3: Update neighbor list for each MS at the BS
  - Step 4: Conduct handover procedures according to the algorithms specified in Section 5.3 if the MS is in scanning period.
- *Simulation Parameter*: The simulation parameters referring to [29] are listed in Table 1. For RSSHD, handover is assumed to be performed as soon as RSSI level is updated. One-slot lookahead policy (*T*_0_ = 1) is employed for PADW in these simulations.

**Table 1:** Simulation parameter summary.

Simulation parameters	Values
#MS, #MBS, and #FBS	90, 2, and 20
Power of MS, MBS, and FBS	13 dBm, 43 dBm, and 13 dBm
Minimum RSSI	-85 dBm
Noise at receiver	-90 dBm
MBS pathloss	34 + 40 log(d)
FBS pathloss	37 + 30 log(d)
System bandwidth	5 MHz
RSSI hysteresis	4 dBm
Throughput model	Shannon capacity
Average moving speed	4 km/h
Handover disruption	100 ms
Parameter in RSSHD, PHD, TPHD and PADW	2 dB-20 dB, 1 m-30 m, 0.01 - 0.1, *ϵ* = 0.05 s - 0.5 s [2]

### 6.2 Simulation results

#### 6.2.1 The effect of handover frequency on throughput

**Fig 5:**
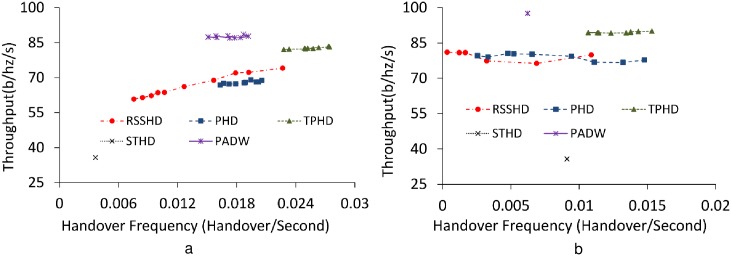
Throughput performance vs. handover frequency with varying hysteresis. a: Free random walk on the map. b: Random walk inside a small area.

We evaluate the performance of the different methods under various values of their hysteresis and parameters listed in Table 1. In Fig 5 depicted by means of the Pareto curves, we show the effect of handover frequency on throughput with parameters of each method varying. In Fig 5(a) we simulate the situations where the mobile users in a random walk move freely in the map (Fig 4). For PADW the locations of the mobile users are predicted by calculating RSSI values with default prediction error margins.

While the handover frequency of STHD is minimum, its throughput is very low. This is because the handover frequency is the only criterion considered for selection. Meanwhile, the handover frequency of RSSHD somewhat varies over the adjustment of hysteresis values, while its average throughput is around 70 (b/hz/s). For example, at the large values such as a large hysteresis margin or long handover delay timer, RSSHD provides quite a low handover frequency since the handovers are deferred. This means that this handover frequency gain is acquired with the cost of throughput degradation. In the result, its handover frequency mainly depends on the hysteresis which is not easily optimized in real situations. Likewise, if distance hysteresis is large enough, the handover frequency and throughput of PHD is low. This is due to the following common principle of two methods: the distance factor also relies on the RSSI level. On the contrary, PADW shows the lower handover frequency than TPHD (40% in average), and keeps it stable at around 0.017 (from 0.015 to 0.02 in Fig 5(a)). This handover frequency of 0.017 is close to average value when compared with those of RSSHD and PHD. Besides, PADW attains the highest throughput performance among all.

In Fig 5(b) the ping-pong effect is examined on the questioning methods when the mobile users move randomly in a small area of 20 meter radius. TPHD shows a high handover frequency. In case of RSSHD, its performance largely relies on the hysteresis as in the free random walk simulation. STHD shows a notably higher handover frequency in this free-walk scenario because it is difficult that the frequent changes of MS’s direction are reflected on the calculation of potential sojourn time of a BS. On the other hand, PADW shows that its handover frequency is relatively low and stable at 0.006 while its throughput gain is the largest among all. In Fig 5(a) and 5(b) PADW attains better performance because PADW determines the association based on the expected reward values in use of predicted locations of MSs, Thus, disturbance caused by stochastic movement is reduced.

#### 6.2.2 Impact of varying the number of FBSs

**Fig 6:**
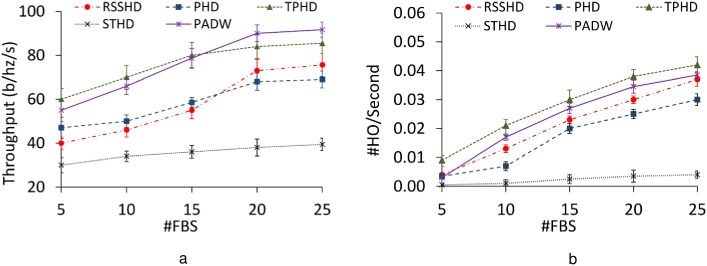
Performance with varying FBS numbers. a: Throughput. b: Handover frequency.

Since the throughput and handover frequency strongly depend on the density of network, we also investigate the performance of these methods with varying the number of FBSs as in Fig 6. Obviously, the throughput and handover frequency increase when increasing the number of FBSs: the MS has more chances to associate to the new FBS which provides better throughput than the current serving FBS. The interesting result is that when the number of FBSs is small, TPHD achieves better throughput than PADW as in Fig 6(a). This slightly better throughput of TPHD justifies the higher handover frequency in Fig 6(b).

On the other side, when the number of the FBSs is high, PADW outperforms other methods. The reason is that in dense environment, the expected communication time between the MS and FBS is reduced. Therefore, the disruption time and mobility have a strong effect on the throughput of the MS. Since PADW considers the handover disruption time and the mobility prediction of the MS as the decision metrics, it can achieve the best throughput among the methods. However, when comparing the throughput in case that the number of the FBSs is 20 and 25, we observe that increasing the number of FBSs does not always lead increases in the throughput. The throughput becomes saturated. Such behaviors are consistent with the result in [1].

#### 6.2.3 Impact of varying the number of MSs

**Fig 7:**
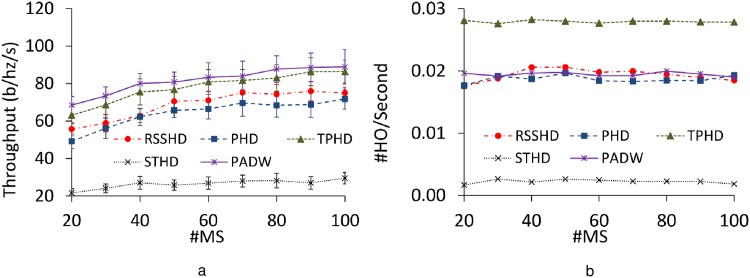
Performance with varying MS numbers. a: Throughput. b: Handover frequency.

We investigate considered methods under various MS populations with default hysteresis. Fig 7 charts out the performance of each method with varying the number of MSs. Although the number of MSs increases, each method shows slightly enhanced throughput. This result is explained as following: The diversity gain is proportional to the population of the MSs. The more the MSs utilized the capacity of FBSs, the higher the network performance becomes. Despite of the presence of a large number of MSs the handover frequency is not affected (Fig 7(b)). Thus, these methods, including PADW, are scalable with respect to network sizes. Furthermore, relative difference between the performances of PADW and the other schemes is stable regardless of the number of fluctuating MSs as well.

#### 6.2.4 Impact of varying MS speed

**Fig 8:**
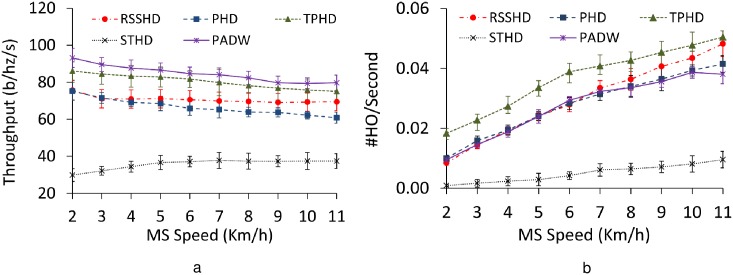
Performance with varying MS speeds. a: Throughput. b: Handover frequency.

We explore how the questioning methods differ from each other when varying MS speed (Fig 8). Handover frequencies certainly increase with the moving speed increasing. This trend comes from the influence of the rising speed. In this tendency, MSs in use of STHD handover to the BS with the longest sojourn time and experience less occurrence of handovers in comparison with the other methods. Therefore, the moving speed has a less effect on STHD. As to throughput, STHD results in a slight increment when the speed is in the range from 2 km/h to 6 km/h. This phenomenon also appears at higher speed (from 7 km/h to 12 km/h), stable around 37 b/hz/s.

On the contrary, the throughput of PADW and TPHD obviously decreases at every speed level, while handover frequency increases in rise of MS speeds. This tendency is due to delayed updates of association states of PADW and TPHD because both methods use multiple handover criteria. However, in case of RSSHD and PHD, throughput is less affected by the high speeds since their association states are kept updated as long as the RSSI level or distance is feasible for the selection of BSs. In spite of this tendency, in general, at average walking speeds (up to 10 km/h), the throughput of PADW is the highest and its handover frequency is relatively comparable to RSSHD and PHD. Even at higher speeds, more than 12 km/h, PADW performs well by enabling the MS to connect to MBSs for maximizing its reward.

#### 6.2.5 Impact of prediction error

**Fig 9:**
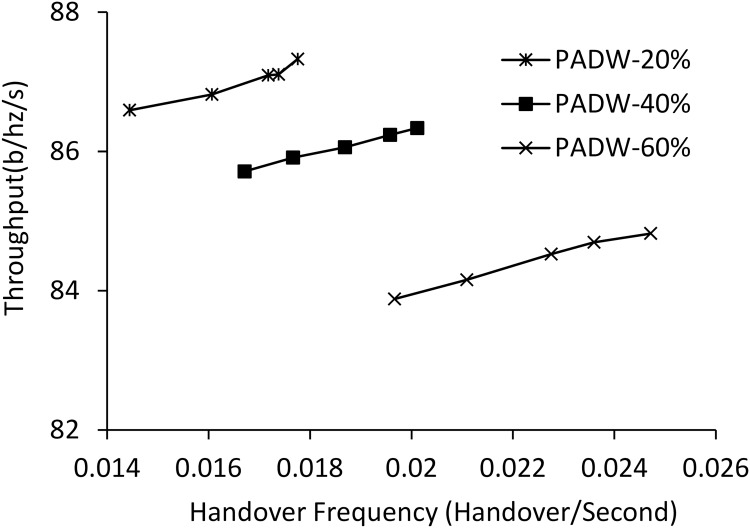
Throughput and handover frequency with varying prediction errors.

We conduct the evaluation of PADW for the effects of erroneous prediction of location on how PADW runs. Fig 9 illustrates the PADW performance when varying the error margins of MSs. Specifically, we apply the different margin rates of 20%, 40% and 60% of the coverage of femtocell. These errors in the location prediction largely increase the handover frequency of PADW. Meanwhile, the throughput remains relatively unchanged and stable despite fluctuating error margins. However, even at the error margin of 40%, the handover frequency of PADW is smaller than one of TPHD around 30% and relatively comparable with that of RSSHD and PHD with hysteresis varying (Fig 5(a)).

When we compare Figs 5(a) and 9, with the error margin of 60%, PADW shows better throughput performance than the other methods. These evaluation results mean that when the movement of the MS can be predicted over very short time period, the errors in the location prediction have more effects on the handover frequency than the throughput. Thus, the erroneous predictions may have more critical impact on streaming applications than best-effort ones. Consequently, the prediction error less than 40% is acceptable, because the performance gain of PADW in terms of handover frequency still remained competitive in comparison with RSSHD and PHD.

#### 6.2.6 Impact of varying minimum throughput threshold

**Fig 10:**
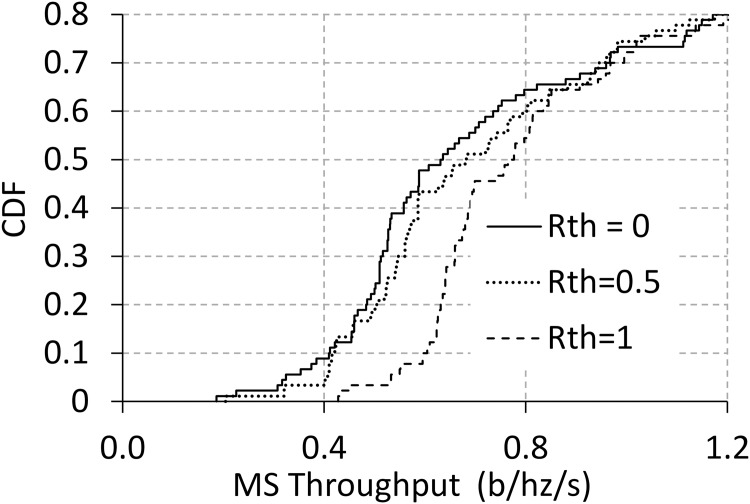
Performance with varying throughput parameters.

In the last scenarios, we investigate the ability of throughput guarantees in PADW as explained in Remark 2. The minimum throughput threshold, i.e. *R*_*th*_, is 0, 0.5, and 1 (b/hz/s), respectively. Fig 10 presents the cumulative distribution function (CDF) of the throughput performance of MSs. When the threshold level increases, the CDF of PADW is pushed toward the right. The increment of the threshold is led to higher values of minimal achievable throughput. However, it is important to notice that this threshold does not guarantee the throughput of every MS. It is a kind of adaptive parameters for satisfying the minimum achievable throughput of MSs.

However, when increasing the threshold level of the minimum throughput of MSs, we obtain a slight decrease in the throughput but a slight increase in handover frequency (Table 2). The situation is explainable, as follows: Imposing more constraints on the association decision results in more occurrences of handovers to guarantee the minimum throughput level. Consequently, guaranteeing the required throughput needs costs of increasing the handover frequency.

**Table 2:** Performance vs. threshold parameters.

*R*_*th*_	0	0.5	1
MS throughput	0.9796	0.9650	0.9415
Handover frequency	0.0186	0.0201	0.0226

From above evaluations, we have following observations: (1) The throughput of PADW is above remaining methods including TPHD which has been declared near optimal [9]. (2) The performance gain of PADW is stable when varying MS speeds and FBS and MS numbers. (3) The handover frequency of PADW is stable under various disruption periods. (4) With large error margins of predictions, handover frequency gain remains significant when compared with TPHD. (5) PADW is able to satisfy the minimum required throughput for MSs by controlling the throughput threshold, *R*_*th*_.

## 7 Conclusions

Dense femtocell networks provide better throughput and extend coverage areas for mobile users. However, the deployment of the large number of femtocell base stations results in more frequent handovers to moving MSs which have an effect on the throughput of the MSs. In this paper we have presented the formal formulation of association control problem regarding to achievable throughput, handover frequency, and mobility prediction of MSs in dense femtocell networks. The association control problem is considered as the dynamic programming problem. In these formulations the term ‘femtocell’ is extended to larger cell sizes encompassing picocells and microcells.

We also introduce the approximate offline algorithm, and derive the low-complexity algorithm, namely online algorithm based on lookahead policy. In addition, we showed that the proposed online algorithm can be distributively implemented at each MS and have performance gains in terms of both throughput and handover frequency when compared with existing schemes. More specifically, the proposed algorithm enhances the throughput while keeping its handover frequency stable and competitive. The remarkable of the proposed algorithm is that it uses ‘effective throughput’ as the decision metric instead of the achievable throughput in previous studies. Note that all the MSs are scheduled following the proportional fair scheduling.

In the future, we plan to investigate the impact of the scheduling algorithms on the association control problem. In more detail, we jointly optimize the scheduling and association problems to improve network-wide performance

## Supporting information

S1 Dataset All the data set for simulation are provided in the S1 Dataset file at https://figshare.com/articles/Dataset_XLSX/4633960. The detail of the data set are as follows: Fig 5 sheet: Throughput performance vs. handover frequency with varying hysteresis (Fig 5)Fig 6 sheet: Performance with varying FBS numbers (Fig 6)Fig 7 sheet: Performance with varying MS numbers(Fig 7)Fig 8 sheet: Performance with varying MS speeds (Fig 8)Fig 9 sheet: Throughput and handover frequency with varying prediction errors (Fig 9)Fig 10 sheet: Performance with varying throughput parameters. (Fig 10).
(XLSX)
